# Multimodal resting-state connectivity predicts affective neurofeedback performance

**DOI:** 10.3389/fnhum.2022.977776

**Published:** 2022-09-08

**Authors:** Lucas R. Trambaiolli, Raymundo Cassani, Claudinei E. Biazoli, André M. Cravo, João R. Sato, Tiago H. Falk

**Affiliations:** ^1^Basic Neuroscience Division, McLean Hospital–Harvard Medical School, Belmont, MA, United States; ^2^McConnell Brain Imaging Centre, Montreal Neurological Institute, McGill University, Montreal, QC, Canada; ^3^Center for Mathematics, Computing and Cognition, Federal University of ABC, São Bernardo do Campo, Brazil; ^4^School of Biological and Behavioural Sciences, Queen Mary University of London, London, United Kingdom; ^5^Big Data, Hospital Israelita Albert Einstein, São Paulo, Brazil; ^6^Institut National de la Recherche Scientifique, University of Quebec, Montreal, QC, Canada

**Keywords:** neurofeedback, brain connectivity, electroencephalography, functional near-infrared spectroscopy, resting-state

## Abstract

Neurofeedback has been suggested as a potential complementary therapy to different psychiatric disorders. Of interest for this approach is the prediction of individual performance and outcomes. In this study, we applied functional connectivity-based modeling using electroencephalography (EEG) and functional near-infrared spectroscopy (fNIRS) modalities to (i) investigate whether resting-state connectivity predicts performance during an affective neurofeedback task and (ii) evaluate the extent to which predictive connectivity profiles are correlated across EEG and fNIRS techniques. The fNIRS oxyhemoglobin and deoxyhemoglobin concentrations and the EEG beta and gamma bands modulated by the alpha frequency band (beta-*m*-alpha and gamma-*m*-alpha, respectively) recorded over the frontal cortex of healthy subjects were used to estimate functional connectivity from each neuroimaging modality. For each connectivity matrix, relevant edges were selected in a leave-one-subject-out procedure, summed into “connectivity summary scores” (CSS), and submitted as inputs to a support vector regressor (SVR). Then, the performance of the left-out-subject was predicted using the trained SVR model. Linear relationships between the CSS across both modalities were evaluated using Pearson’s correlation. The predictive model showed a mean absolute error smaller than 20%, and the fNIRS oxyhemoglobin CSS was significantly correlated with the EEG gamma-*m*-alpha CSS (*r* = −0.456, *p* = 0.030). These results support that pre-task electrophysiological and hemodynamic resting-state connectivity are potential predictors of neurofeedback performance and are meaningfully coupled. This investigation motivates the use of joint EEG-fNIRS connectivity as outcome predictors, as well as a tool for functional connectivity coupling investigation.

## Introduction

During a neurofeedback session, participants are trained to achieve volitional control of their neural activity through real-time feedback (Sitaram et al., 2017). Potential applications of this approach in psychiatry include the treatment of mood and anxiety disorders, such as depression (Trambaiolli et al., 2021a) and obsessive-compulsive disorder (OCD) (Ferreira et al., 2019). Clinical benefits have been reported to persist for weeks or months after training (Rance et al., 2018). However, not all users will benefit from neurofeedback training, and it is a current interest in this research field to identify potential predictors of performance and outcomes (Weber et al., 2020; Haugg et al., 2021). Recently, targeted functional connectivity has been explored as a potential predictor of neurofeedback training performance. This has been motivated by findings showing that individual differences in resting-state functional connectivity can identify individuals from a large group (Finn et al., 2015), and influence task performances in different cognitive domains (Shen et al., 2017). For example, Scheinost et al. (2014) showed that fMRI-based whole-brain functional connectivity could be used as a predictor of affective neurofeedback treatment success in patients with OCD (Scheinost et al., 2014).

However, for a clearer picture of the neurobiological mechanisms underlying the relations between resting-state networks and task outcomes, a better understanding of the neural activity related to spontaneous hemodynamic fluctuations is required (Ma et al., 2016). In this context, several studies have explored the combination of electrophysiological and hemodynamic methodologies (Ingvar et al., 1979; Okyere et al., 1986; Goldman et al., 2002; Mantini et al., 2007; Scheeringa et al., 2008; Britz et al., 2010; Schölvinck et al., 2010; Yuan et al., 2012; Talukdar et al., 2015). A previous study combining functional magnetic resonance imaging (fMRI) blood oxygen level-dependent (BOLD) signals with local field potential records in monkeys observed correlated spontaneous fluctuations over the cortex, particularly for gamma-band power (Schölvinck et al., 2010). In humans, the neurovascular coupling has been broadly studied by combining the fMRI-BOLD signal with electroencephalography (EEG) recordings (Goldman et al., 2002; Mantini et al., 2007; Scheeringa et al., 2008; Britz et al., 2010; Yuan et al., 2012). An inverse correlation between the BOLD signal amplitude and the EEG alpha frequency power has been consistently observed (Goldman et al., 2002). Moreover, a linear correlation between the EEG spectro-temporal amplitude modulation (EEG-AM) and the blood flow through the gray matter has also been observed (Ingvar et al., 1979; Okyere et al., 1986; Talukdar et al., 2015; Trambaiolli et al., 2020).

Nevertheless, simultaneous EEG-fMRI recording is challenging. The EEG signal is corrupted by magnetic gradient artifacts due to interference caused by the MRI, and artifacts on functional images can be caused by the electrodes positioning (Talukdar et al., 2015). Recently, functional near-infrared spectroscopy (fNIRS) has emerged as a useful tool combined with EEG since there is no electro-optical interference (Talukdar et al., 2015; Chiarelli et al., 2017). Moreover, fNIRS data provides information about oxyhemoglobin and deoxyhemoglobin concentration changes at the cortical surface with relatively high sampling rates (Strangman et al., 2002; Cui et al., 2011), allowing for the temporal evaluation of neurovascular coupling. Joint EEG-fNIRS measurement was recently applied to several neuroscientific questions, such as to investigate the neurodevelopment (Roche-Labarbe et al., 2007), language processing (Wallois et al., 2012), or to develop brain-computer interfaces (BCI) (Fazli et al., 2012; Tomita et al., 2014; Banville et al., 2017).

In this study, we combined EEG and fNIRS data with a task-related connectivity modeling (Finn et al., 2015; Shen et al., 2017) to predict participants’ performance in an fNIRS-based affective neurofeedback task (Trambaiolli et al., 2018). The interest in this particular task is due to the potential of fNIRS-based protocols to be used as an intervention outside the overcontrolled environment of laboratories (Trambaiolli et al., 2021b). We achieved a satisfactory performance prediction using these modalities as inputs for a support vector regressor. We also identified that connectivity profiles derived from the EEG-AM and fNIRS hemoglobin concentrations are significantly correlated. These results suggest that complementary neurovascular signatures converge to the same task-preparation network to perform best in the upcoming neurofeedback task.

## Materials and methods

### Participants

Thirty-one healthy participants (16 women), aged between 20 and 35 years (mean age of 25.71 ± 3.32 years) and all undergraduate or graduate students were recruited. The subjects had no diagnosis of neurological diseases (ICD-10: G00G99) or psychiatric disorders (ICD-10: F00-F99) and had a normal or corrected-to-normal vision. Ethical approval was obtained from the Ethics Committee of the Federal University of ABC, and all participants provided written consent before participation.

### Data acquisition

**FIGURE 1:**
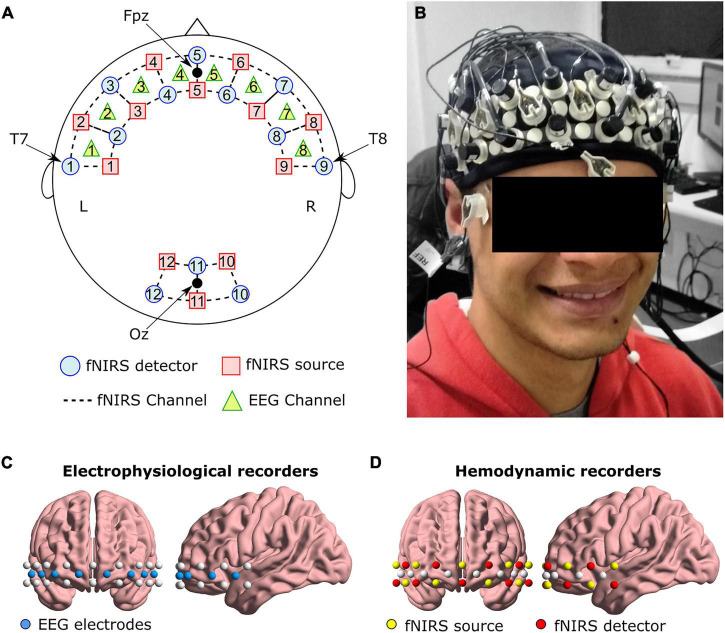
**(A)** Schematic representation of EEG and fNIRS channels, and **(B)** an example of the cap on one volunteer. Anatomical representation of the positioning of **(C)** EEG electrodes, and **(D)** fNIRS optodes.

fNIRS recording was performed using the NIRScout System (NIRx Medical Technologies, LLC. Los Angeles, California) with an array of 11 light sources and 11 detectors. These optodes were arranged in an elastic cap, with 9 pairs of source-detectors positioned over the fronto-temporal regions and 3 pairs of source-detectors over the occipital region as depicted in Figures 1A,B,D. We adopted four positions from the international 10–20 EEG system as reference points during the setup: detectors 1 and 9 were positioned approximately over the T7 and T8 positions, respectively, while the Fpz and Oz were in the center of channels 5-5 and 11-11, respectively. Source-receptor distance was 30 mm for contiguous optodes, and the used wavelengths were 760 and 850 nm. This setup resulted in a total of 32 source-detector channels. Signals obtained from these 32 channels were measured with a sampling rate of 5.2083 Hz using the NIRStar 14.0 software (NIRx Medical Technologies, LLC. Los Angeles, California).

Eight active EEG channels (see Figures 1A–C) were recorded using a 72-channel QuickAmp amplifier system (Brain Products GmbH, Germany). To ensure that any lateralization effect was not caused by the reference placement, for half of the subjects the reference electrode was positioned over the left earlobe and the ground electrode over the right earlobe. For the second half, these positions were inverted (reference on the right earlobe and ground on the left earlobe). The BrainVision Recorder software (Brain Products GmbH, Germany) was used to acquire the data with a sampling frequency of 500 Hz and without applying filters during acquisition.

Electrode and optode locations followed the same placement as in the original neurofeedback experiment (Trambaiolli et al., 2018). The frontal cortex includes important regions for emotion processing (e.g., the orbitofrontal cortex and the lateral and medial prefrontal cortex) (Lindquist et al., 2012, 2015) and is relevant during the affective neurofeedback task.

### Neurofeedback task

All volunteers participated in an affective neurofeedback task (Trambaiolli et al., 2018) that consisted of 5 min of continuous eyes-opened resting-state, followed by two pairs of training-test blocks with 10 and 11 trials, respectively. During the training and test blocks, subjects were instructed to remember autobiographical memories with a positive affect context or to remain relaxed (rest with eyes opened), depending on the stimuli presented on the screen. For each trial, a 3-s moving window extracted average values of oxyhemoglobin and deoxyhemoglobin concentrations to compose the respective feature set. Data from each training block was used to train a linear discriminant analysis (LDA) model, which was used to provide feedback to the participant during each test block. The “performance” measure used in the next steps corresponds to the number of trials during the test blocks where the user achieved minimum control of the system. The interested reader is referred to Trambaiolli et al. (2018), where a detailed description of the protocol and the original report of the results from the neurofeedback task are provided.

### Electroencephalography pre-processing

#### Filtering and artifact correction

First, the pre-task resting block was segmented from the raw data and then band-pass filtered between 0.1 and 100 Hz by a second-order Butterworth filter. Then, we applied the wavelet-enhanced independent component analysis (wICA) for artifact correction (Castellanos and Makarov, 2006; Akhtar et al., 2012). With this method, EEG signals were decomposed into independent components (ICs), and the discrete wavelet transform (DWT) was applied to each IC. In sequence, wavelet thresholds were selected to differentiate between neural and artifactual coefficients. The inverse wavelet transform was applied to these thresholds, retrieving only data related to neural activity in each IC. Importantly, the wICA method allows for recovering neural data leaked on noisy ICs by filtering out artifacts from the wavelet-decomposed signals in each IC (Castellanos and Makarov, 2006). Thus, no IC exclusion was necessary. Lastly, the artifact-free EEG data were reconstructed using the wavelet-corrected ICs (Castellanos and Makarov, 2006; Akhtar et al., 2012). All these steps were performed using the EEGLAB (Delorme and Makeig, 2004) and wICA toolboxes (Castellanos and Makarov, 2006), with the cleaning artifact tolerance set to 1.4 and the IC artifact detection threshold to 1.6. Both values were empirically determined.

#### Amplitude modulation computation

Amplitude modulations were computed following the procedure described in Trambaiolli et al. (2011, 2020) and Falk et al. (2012). Briefly, the full band temporal series from all signals were decomposed into five classical spectral bands, namely: delta (0.1–4.0 Hz), theta (4.0–8.0 Hz), alpha (8.0–12.0 Hz), beta (12.0–30.0 Hz), and gamma (30.0–50.0 Hz), using finite impulse response (FIR) bandpass filters. The temporal envelope was extracted from each frequency band using a Hilbert transform. Then, each envelope was further decomposed into five modulation bands called: *m*-delta (0.1–4.0 Hz), *m*-theta (4.0–8.0 Hz), *m*-alpha (8.0–12.0 Hz), *m*-beta (12.0–30.0 Hz), and *m*-gamma (30.0–50.0 Hz), using FIR bandpass filters. However, due to Bedrosian’s theorem (Bedrosian, 1963), the envelope signal can only contain modulation frequencies up to the maximum frequency of its originating signal. Hence, if we use the notation “frequency band—*m*—modulation band,” only the following amplitude modulations are relevant: delta-*m*-delta, theta-*m*-delta, theta-*m*-theta, alpha-*m*-delta, alpha-*m*-theta, beta-*m*-delta, beta-*m*-theta, beta-*m*-alpha, beta-*m*-beta, gamma-*m*-delta, gamma-*m*-theta, gamma-*m*-alpha, gamma-*m*-beta, and gamma-*m-*gamma.

As previous studies have reported that alpha modulations are related to the regional blood flow (Ingvar et al., 1979; Okyere et al., 1986; Jann et al., 2010; Trambaiolli et al., 2020), the present study focused exclusively on the alpha band modulations. In other words, we used for further analysis the beta-*m*-alpha and gamma-*m*-alpha modulations.

#### Electroencephalography connectivity matrices

For each of the previously mentioned alpha modulation bands, the connectivity between two channels was measured using Pearson’s correlation over the whole pre-task resting block (Bastos and Schoffelen, 2016). All possible combinations of channels were used to generate a connectivity matrix of 8 × 8 dimensions (a total of two EEG-based connectivity matrices per participant: one for beta-*m*-alpha and one for gamma-*m*-alpha).

### Functional near-infrared spectroscopy pre-processing

#### Filtering and hemoglobin concentrations computation

The fNIRS data were pre-processed using the NIRStar toolbox (v. 14.0, NIRx Medical Technologies, LLC. Los Angeles, California). First, fNIRS raw data were bandpass filtered between 0.01 and 0.2 Hz by a linear-phase FIR filter. Then, the filtered temporal series were detrended by their respective whole-length record (without segmentation). Next, the oxy and deoxyhemoglobin concentrations were computed using the modified Beer-Lambert law, with the differential pathlength factor (DPF) set to 7.25 and 6.38, respectively, for both fronto-temporal and occipital regions (Essenpreis et al., 1993). Finally, the pre-task resting-state block was segmented for the functional connectivity analysis.

#### Functional near-infrared spectroscopy connectivity matrices

For each hemoglobin concentration preprocessed time series, the functional connectivity between two channels was measured using Pearson’s correlation over the whole pre-task resting block (Bastos and Schoffelen, 2016). Considering the disposition of all fNIRS and EEG channels in our experiment, only the 25 fNIRS channels around the EEG electrodes (Figure 1) were used to compute the connectivity. Hence, each hemoglobin concentration resulted in a connectivity matrix of 25 × 25 dimensions (a total of two fNIRS-based connectivity matrices per participant: one for oxyhemoglobin and one for deoxyhemoglobin).

### Connectivity predictive modeling

#### Connectivity profiles

**FIGURE 2:**
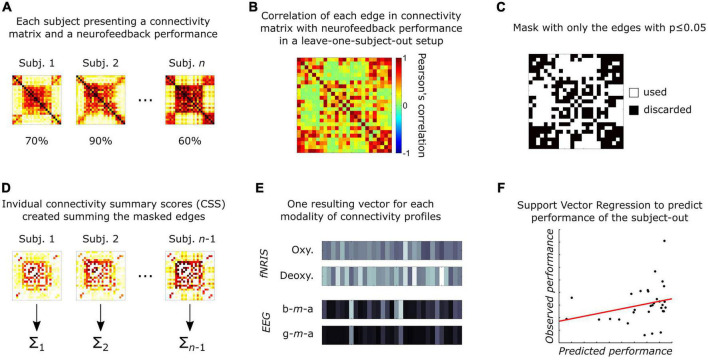
Schematic representation of the connectivity-based predictive modeling (adapted from Shen et al., 2017), summarized as: **(A)** Each participant has one connectivity matrix per neuroimaging feature and one performance measurement related to the affective neurofeedback task; **(B)** each edge in connectivity matrices is related to the subject’s performance using Pearson’s correlation in a leave-one-subject-out (LOSO) setup; **(C)** next, significant edges (*p* ≤ 0.05) are selected to create a mask; **(D)** then, for each subject in the training group, the mask is applied to select most important edges, which are then summarized (summed) into a single CSS value per subject; **(E)** next, an inter-subject vector is created for each neuroimaging modality, containing the respective summary values; **(F)** finally, these four vectors are used to train a support vector regressor (SVR) and test it in the subject-out.

Up until now, all analyses were performed using data from each subject at a time. Now, the next steps focus on the inter-subject approach and follow the connectome-based predictive modeling method proposed by Shen et al. (2017) (see Figure 2). These steps were repeated for each connectivity matrix.

In our analysis, we used a leave-one-subject-out (LOSO) setup, as it emulates how the prediction of performance would happen when evaluating new patients in real-world scenarios. For each LOSO iteration, each edge of the connectivity matrices (from all subjects but one, i.e., training group) was correlated with their performance during the neurofeedback task, resulting in a correlation matrix that was used to create a mask consisting of all edges presenting significant correlations (*p* ≤ 0.05). This mask was then applied to the original connectivity matrices of each subject from the training group. The remaining edges were summed, creating a single connectome value (here called “connectome summary score”—CSS) for each subject. This procedure was repeated for each modality (two from fNIRS—oxyhemoglobin and deoxyhemoglobin—and two from EEG—beta-*m*-alpha and gamma-*m*-alpha). As result, four CSS values were obtained per subject, and these were used as features to train a linear support vector regressor (SVR) to predict the performance during the neurofeedback task. The hyperparameter C was set to 1, and epsilon was estimated as a tenth of the standard-deviation of the performance values in the training set. Finally, the SVR was then tested in the left-out subject. These steps were repeated, each time leaving a different subject out until we tested all possible setups.

#### Correlation comparison

To evaluate intra- and inter-modality correlations, we calculated the two CSS vectors for each modality using edges selected in at least one LOSO iteration. Then, we computed Pearson’s correlation with these vectors. In intra-modality analyses, for fNIRS it was oxyhemoglobin vs. deoxyhemoglobin, and for EEG beta-*m*-alpha vs. gamma-*m*-alpha. For inter-modality analyses, we explored oxyhemoglobin vs. beta-*m*-alpha, oxyhemoglobin vs. gamma-*m*-alpha, deoxyhemoglobin vs. beta-*m*-alpha, and deoxyhemoglobin vs. gamma-m-alpha. Then, for each combination, we performed 10^6^ permutations of CSS values across subjects to establish a confidence interval. Finally, the resulting *p*-values were corrected using the false discovery rate (FDR) method for six multiple comparisons.

## Results

### Connectivity-based prediction of performance

**FIGURE 3:**
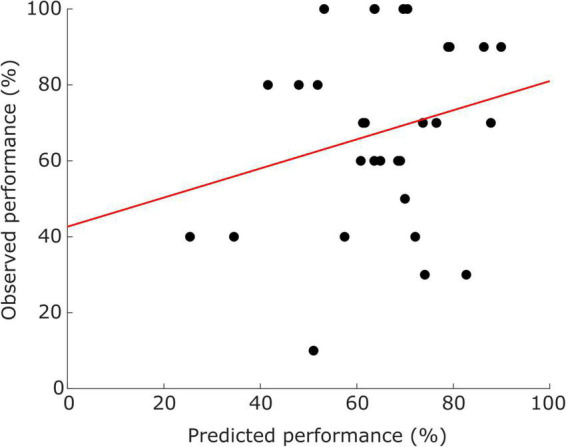
Scatter plots illustrating the distribution of participants (dots) according to predicted performance (*x*-axis) and observed performance (*y*-axis). Red line represents the trend.

A detailed description of the neurofeedback performance is reported in Trambaiolli et al. (2018). In the current study, we only considered the performance in trials with real feedback from the original experiment. The included sample presents different levels of neurofeedback literacy, ranging from 0 to 100% of accuracy, with a median ± standard-deviation of 70.00 ± 24.18%, The performance prediction combining the CCS values from both modalities (four features) and the SVR showed a mean absolute error (MAE) of 19.68 ± 15.65%; a scatterplot of the observed vs. predicted performances is shown in Figure 3.

### Multimodal connectivity profiles

**FIGURE 4:**
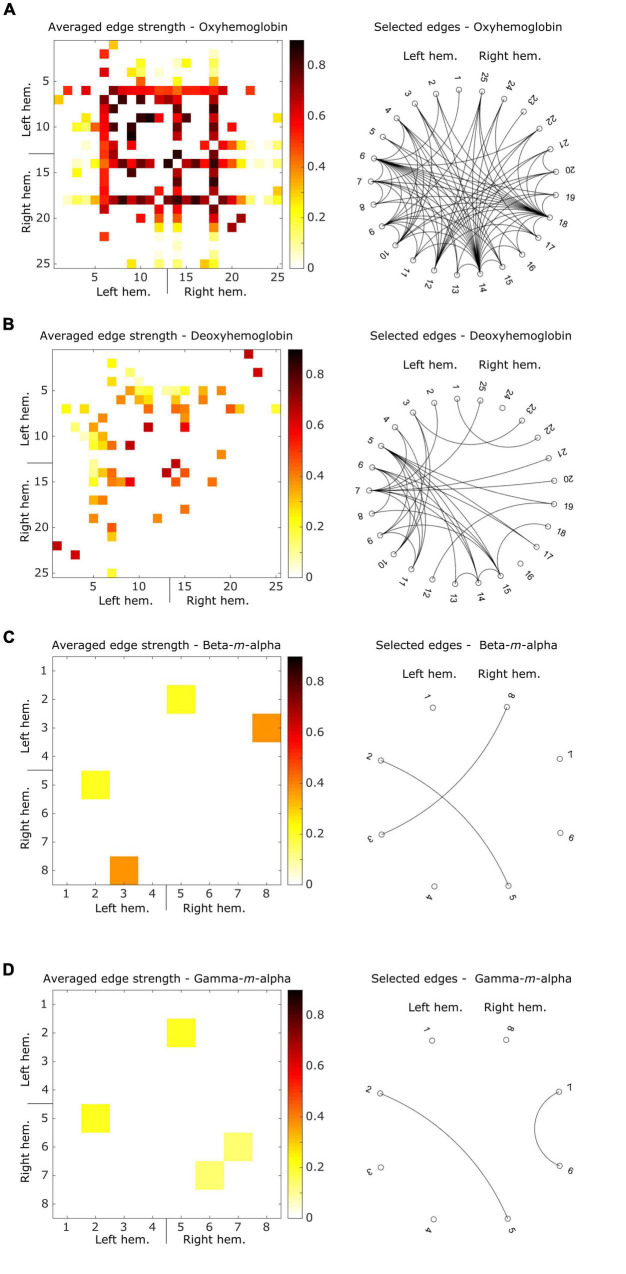
Averaged connectivity matrices showing the strength of the selected edges, and ring graphs describing the binary connections resulting from these edges for **(A)** oxyhemoglobin, **(B)** deoxyhemoglobin, **(C)** beta-*m*-alpha, **(D)** gamma-*m*-alpha. For connectivity matrices, hotter colors represent strong connectivity, while white squares represent connections removed by the correlation-based mask. For ring graphs, each black line represents a binary connection between two areas.

Figure 4 shows ring graphs with the edges selected at least once during the LOSO iterations of the connectivity-based predictive model, as well as matrices with averaged edge strengths. fNIRS oxyhemoglobin (Figure 4A) data showed a total of 82 unique edges selected, with a balanced number of edges in each hemisphere (20 in the left, 22 in the right, and 40 inter-hemispheric). The deoxyhemoglobin connectome (Figure 4B) had a total of 33 edges, with a higher concentration in the left hemisphere (Scheeringa et al., 2008) than in the right one (Trambaiolli et al., 2021a), and 18 inter-hemispheric connections. From the EEG data, both beta-*m*-alpha (Figure 4C) and gamma-*m*-alpha (Figure 4D) had two edges selected each. Beta-*m*-alpha edges were both inter-hemispheric, while the gamma-*m*-alpha connectome had one edge in the right hemisphere and one inter-hemispheric connection.

**FIGURE 5:**
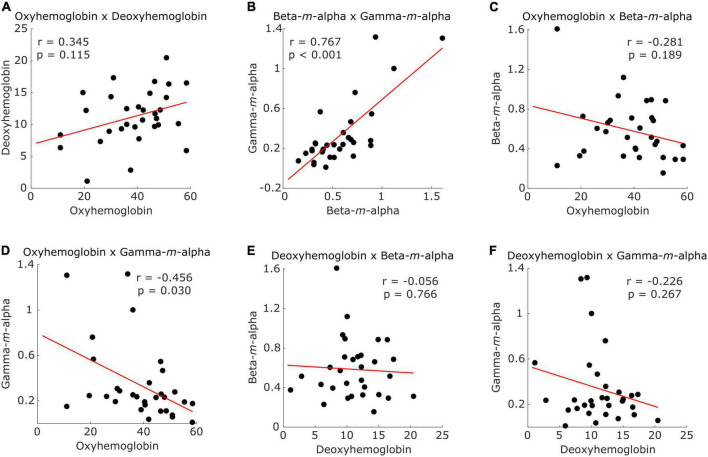
Scatter plots illustrating participants (dots) distribution according to **(A)** CSS from fNIRS oxyhemoglobin and deoxyhemoglobin, **(B)** CSS from EEG beta -*m*-alpha and gamma-*m*-alpha, **(C)** CSS from fNIRS oxyhemoglobin and EEG beta-*m*-alpha, **(D)** CSS from fNIRS oxyhemoglobin and EEG gamma-*m*-alpha, **(E)** CSS from fNIRS deoxyhemoglobin and EEG beta-*m*-alpha, and **(F)** CSS from fNIRS deoxyhemoglobin and EEG gamma-*m*-alpha. Red line represents the trend.

Figure 5 presents the distribution of participants according to different combinations of CSS values. Intra-modality CSS pairs showed a positive correlation for both fNIRS (Figure 5A) and EEG (Figure 5B) combinations, but only EEG-based CSS values showed a significant correlation (*r* = 0.767, *p* < 0.001). Inter-modality pairs showed a negative correlation in all combinations (Figures 5C–F), with a significant correlation between the fNIRS oxyhemoglobin and the EEG gamma-*m*-alpha features (*r* = −0.456, *p* = 0.030).

## Discussion

In this study we evaluated if simultaneous EEG and fNIRS recordings during a pre-task resting condition would provide information about subsequent neurofeedback performance and present convergent connectivity profiles. This approach revealed good prediction accuracy and a significant correlation between the CSS values from EEG gamma-*m*-alpha modulation band and fNIRS oxyhemoglobin concentration. Moreover, both pre-task resting-state matrices presented similar clusters of intra and inter-hemispheric functional connections.

Our results are consistent with a recent trend of investigations for neurofeedback performance predictors (Alkoby et al., 2018; Weber et al., 2020). Previously, resting-state functional connectivity showed a good prediction of performance, or outcomes, in neurofeedback or brain-computer interface tasks when calculated using fMRI (Scheinost et al., 2014), EEG (Zhang et al., 2013, 2015; Lee et al., 2020), or fNIRS data (Trambaiolli et al., 2018) individually. Complementary to region-specific or psychophysiological predictors (Weber et al., 2020), connectivity-based predictors provide network-wide information about the neural mechanisms involved in the control of the proposed protocol. These could include information about resting-state networks involved in preparation for the neurofeedback training or those specific to the task (e.g., emotion processing networks for affective neurofeedback or motor control network for motor imagery neurofeedback). However, although the combination of hemodynamics and electrophysiological measures is becoming a common approach for the development of neurofeedback protocols (Perronnet et al., 2017), this is the first time that they are combined as a neurofeedback performance predictor. Thus, we present an innovative perspective for future experiments aiming to understand the neural basis of neurofeedback success.

Importantly, new EEG and fNIRS equipment are portable and allow quick setup when using dry and active electrodes (Lopez-Gordo et al., 2014) or modular multimodal sensors (von Lühmann et al., 2016). Thus, these imaging modalities are ideal for naturalistic neurofeedback implementations (Trambaiolli et al., 2021b). Moreover, the proposed pipeline is simple and has low computational cost. These characteristics make the proposed protocol a promising tool for real-world applications. For instance, affective neurofeedback shows encouraging results in treating depression (Trambaiolli et al., 2021a). This connectivity-based predictor could be used in the future to select personalized treatments, identifying patients who would potentially benefit from affective neurofeedback and those who would benefit from other treatment options.

Regarding intra-modality CSS correlations, it is notable that both pairs of modalities presented a positive correlation between features. The significant result between EEG-based features can be explained by the fact that both EEG-AM frequencies are modulated by the same modulation band. The EEG-AM analysis consists of the spectral characterization of the temporal changes of the instantaneous amplitude of the EEG signals. Thus, it provides information on the second-order periodicities (sometimes referred to as “hidden periodicities”), i.e., modulation frequencies, which are present in the EEG signals (Cassani and Falk, 2018). These frequency interactions have a fundamental importance in neural communication since this conjunction creates different neural signatures with the integration of activity across different temporal and spatial scales (Canolty and Knight, 2010; Hyafil et al., 2015). On the other hand, the non-significant result between fNIRS-based CSS might be related to the difference in the number of edges selected for each concentration. Also, oxyhemoglobin and deoxyhemoglobin concentrations present a high negative correlation (Cui et al., 2010), and the functional connectivity analysis resulting from these measures might present different distributions over the scalp (Sasai et al., 2011).

The oxyhemoglobin and deoxyhemoglobin time series are spatially and temporally correlated with the superficial fMRI-BOLD signal (Strangman et al., 2002; Huppert et al., 2006; Toronov et al., 2007; Cui et al., 2011; Sato et al., 2013). They show similar connectivity results to those measured by regional fMRI signals close to the fNIRS channels (Sasai et al., 2012). Thus, convergence between fNIRS and EEG-based connectivity profiles is expected for features related to neurovascular coupling phenomena (i.e., the local hemodynamic changes driven by regional neural activity) (Shibasaki, 2008). For example, EEG and BOLD-related functional connectivity are related to similar phenotypes (Nentwich et al., 2020), and share mutual information (Keinänen et al., 2018; Wirsich et al., 2020). Also, previous studies show that different characteristics of the EEG alpha band (i.e., spectral peak, power, latency, etc.) present a negative relationship with the BOLD signal (Goldman et al., 2002; Laufs et al., 2003; Gonçalves et al., 2006; Horovitz et al., 2008; Jann et al., 2009, 2010; Britz et al., 2010) and BOLD-based functional connectivity (Scheeringa et al., 2012; Chang et al., 2013). On the other hand, EEG gamma frequencies positively correlate with the BOLD signal (Lachaux et al., 2007; Scheeringa et al., 2011), while local field potential gamma frequencies are predictors of the BOLD fluctuation (Niessing et al., 2005; Nir et al., 2007; Viswanathan and Freeman, 2007; Goense and Logothetis, 2008). The opposite relationship between alpha and gamma with BOLD suggests different roles from both frequencies in the brain communication (Scheeringa and Fries, 2019). In fact, the alpha rhythm is described as an active inhibitor that modulates task-irrelevant brain regions (Jensen and Mazaheri, 2010; Zumer et al., 2014), while the gamma cycle reflects the alternation of excitability in groups of neurons to respond to new inputs (Ni et al., 2016). In the context of our pre-task resting-state connectome modeling, both rhythms may be constantly integrated into a transitional loop of inhibitory-excitatory processes while the participant gets ready to perform the neurofeedback task. For example, the gamma-alpha interaction was already described in human neuroimaging, mainly over sensory areas (Osipova et al., 2008; Voytek et al., 2010; Spaak et al., 2012; Bonnefond and Jensen, 2015). This interaction is suggested as a combination of the alpha function while filtering sensory incomes with the gamma role in the active processing information (Bonnefond and Jensen, 2015).

In both modalities, we see a predominance of inter-hemispheric connections selected as relevant edges. Although not linearly dependent nor perfectly spatiotemporally correspondent (Chiarelli et al., 2017), source estimation of EEG and fNIRS signals showed a significant level of corresponding sensitivity to gray matter activity between co-localized channels (Giacometti and Diamond, 2014), which may explain this multimodal consistency. Additionally, prior studies exploring comparable characteristics in fNIRS-EEG recordings reported that the variation of oxyhemoglobin and deoxyhemoglobin concentrations show intra- and inter-areal temporal coupling with the power peaks in EEG alpha frequencies at rest (Koch et al., 2008; Pfurtscheller et al., 2012; Keles et al., 2016), the component amplitude of visually evoked potentials (Obrig et al., 2002), and the whole-head alpha band connectivity during hypercapnia (Babiloni et al., 2014). Also, epidural fNIRS and electrophysiological recordings in anesthetized macaque monkeys reported a temporal correlation between the vascular and neural modulation peaks (Zaidi et al., 2015). These phenomena might be the foundation for the correlated trends between fNIRS and EEG CSS values and the similar predominance of inter-hemispheric connections in both modalities.

Our study presents some limitations which should be considered when interpreting our results. First, fNIRS signals may also encompass peripheral changes [e.g., skin impedance, muscular and cranial blood flow, among others (Nishitani and Shinohara, 2013)]. Although this was not controlled in our design, future studies should consider using short-separation channels to filter systemic hemodynamic fluctuations from non-neural sources (Gagnon et al., 2014; Brigadoi and Cooper, 2015). Similarly, for the EEG connectivity measure, methods for volume conduction correction could be explored to avoid signal leakage (Brookes et al., 2012). Given the wide range of methods to infer functional connectivity, future studies could also evaluate other approaches for connectivity analysis, such as those estimating directional, non-linear, or frequency-domain interactions (Bastos and Schoffelen, 2016). Future studies should also explore the possibility of feeding the regressor model directly with the selected multimodal features instead of summarizing them into CSS values. For this, penalized regression methods, such as ridge regression, could be used (Gao et al., 2019).

Another important point is that our experimental design focused on positioning detectors over the frontal cortex. Thus, relevant brain structures to the resting-state networks, such as the cingulate cortex and the precuneus for the default mode network (Raichle et al., 2001), or the insular cortex, basal ganglia, and amygdala for the salience network (Seeley et al., 2007), are not covered here. However, to the best of our knowledge, there is no pair of electro-hemodynamic modalities capable of recording simultaneously some of these deep brain structures. Other sensor configurations would also be relevant for neurofeedback or BCI task of interest. For instance, central regions could be relevant when predicting the performance of motor imagery protocols (Hwang et al., 2009) and parietal regions for attention-based experiments (Bagherzadeh et al., 2020). Future studies using EEG-fNIRS modalities could also explore whole-brain connectivity using evenly distributed electrodes to evaluate the role of multiple brain regions as performance predictors.

## Conclusion

This paper provided preliminary evidence of the feasibility of multimodal predictors of neurofeedback performance and demonstrated the convergence of resting-state functional connectomes across modalities. Considering the portability and simplicity of EEG and fNIRS modalities, this type of result is promising for a wide range of applications which might be benefited from resting-state connectivity analysis, such as the diagnosis or prediction of treatment effectiveness in neurological and psychiatric disorders. Also, our results suggest the combined use of fNIRS and EEG methods in the investigation and description of the neurovascular coupling phenomena in network neuroscience.

## Data availability statement

The raw data supporting the conclusions of this article will be made available by the authors, without undue reservation.

## Ethics statement

The studies involving human participants were reviewed and approved by the Ethics Committee of the Federal University of ABC. The patients/participants provided their written informed consent to participate in this study.

## Author contributions

LT, JS, and TF conceptualized the experiment. LT and CB collected the data. LT and RC performed the analysis. All authors contributed to the article and approved the submitted version.
